# Effects of Social Participation and Its Diversity, Frequency, and Type on Depression in Middle-Aged and Older Persons: Evidence From China

**DOI:** 10.3389/fpsyt.2022.825460

**Published:** 2022-04-25

**Authors:** Jiahui Wang, Jiao Xu, Yizhen Nie, Pochuan Pan, Xin Zhang, Ye Li, Huan Liu, Libo Liang, Lijun Gao, Qunhong Wu, Yanhua Hao, Saleh Shah

**Affiliations:** ^1^Centre of Health Policy and Management, Health Management College, Harbin Medical University, Harbin, China; ^2^Department of Social Medicine, Health Management College, Harbin Medical University, Harbin, China; ^3^Physical Examination Center, The Second Affiliated Hospital of Harbin Medical University, Harbin, China; ^4^Department of Government Policy and Public Management, Graduate School of Chinese Academy of Social Sciences, Beijing, China

**Keywords:** social participation, depression, propensity score matching, China, elderly

## Abstract

**Background:**

Depression is one of the greatest public health problems worldwide. The potential benefit of social participation (SP) on mental health has been widely acknowledged. Nevertheless, a few studies have used propensity score matching (PSM) to reduce the influence of data bias and confounding variables. This study explored the effect of social participation on depression among middle-aged and older Chinese persons through a PSM method, considering the frequency, type, and quantity of SP. Effects were compared among different age groups, genders, and places of residence.

**Methods:**

The datasets were obtained from the 2018 wave of the China Health and Retirement Longitudinal Study. A total of 9,404 respondents aged 45 and above were included in the study. PSM and ordinary least squares methods were used to estimate the effect of social participation on depression.

**Results:**

PSM estimation results showed that SP had a significantly positive effect on decreasing depression scores (*p* < 0.001) by 0.875–0.898 compared with persons without SP. All types of SP had a significantly positive effect (*p* < 0.001), and participating in community activities had the largest effect (β = −1.549 to −1.788, *p* < 0.001). Higher frequency of participation and more types of SP promoted lower depression scores; subgroup analyses revealed that the promotion effect was significantly greater among women, those aged ≥75 years, and those living in urban areas.

**Conclusion:**

PSM indicated that SP could alleviate the depression of middle-aged and older Chinese persons. Targeted measures should be adopted to promote SP and thereby improve mental health and promote healthy and active aging.

## Introduction

Depression is a common mental disease, which can cause serious harm to physical and mental health if long-term relief from symptoms is not achieved. The widespread impact of depression is not only reflected in the extremely high health lost (1) and numerous complications, such as type 2 diabetes (2) and cardiovascular diseases (3), but also in its promotion of suicide. Depression has evolved into a serious social problem and has accordingly attracted wide research and clinical attention.

According to the latest estimates, more than 300 million people currently experience depression (4), and the prevalence increased by 18.4% between 2005 and 2015. Among different age groups, prevalence peaks in middle and old age (5). With the development of an aging population, depression among middle-aged and older people would become relatively more prevalent. China, as the largest developing country, is also experiencing a trend of rapid population aging. It is estimated that between 2012 and 2050, China's elderly population will increase from 194 million to 483 million, and the aging level will increase from 14.3 to 34.1%; at that time, China would be one of the countries with the fastest population aging rate (6). A study indicated that there were 56.36 million people living with depression in China in 2017, accounting for 21.3% of cases worldwide (7). The prevalence rate of depression increases with age and becomes more serious in persons over 60 years. Thus, for China, it is important to adopt measures to promote mental health among high-risk groups, such as middle-aged and older people, to avoid the adverse outcomes of depression. This would also provide the benefit of globally improving mental health.

Currently, conventional interventions against depression, such as cognitive behavioral therapy and medication, are considered effective approaches. However, it is difficult to meet the needs of low- and middle-income countries for current depression treatments, given resource limitations (8); the cost for depression treatment is notably high. It has been estimated that an annual global cost of US$1.15 trillion is attributable to depression (9). In China, a study calculated that total economic expenditure per capita for depression was 21,650 CNY, with direct economic expenditure of 6,806 CNY and indirect economic expenditure of 14,844 CNY (10). This heavy economic burden on developing countries is not affordable. Thus, it is important to seek other effective and feasible low-cost approaches and pay particular attention to preventive interventions.

Since the concept of SP was introduced to the field of aging research at the beginning of the 20th century (11), increasing attention has been paid to the relationship between SP and depression. Activity theory posits that SP by older persons helps them to maintain self-esteem, obtain psychological satisfaction, and live a long and healthy life. Maintaining intellectual, physical, and certain social activities are necessary in later life, not only to promote physical health but also to foster mental health (12, 13). Prior studies also indicated the relationship between SP and depression among the elderly group. A study found that SP plays a protective role against depressive symptoms (14), and continuous SP may have strong association with fewer depressive symptoms (15). Moreover, a few studies further explored the effect of different types of SP on depression. Formal voluntary activities reduce the risk of depression in older persons, but informal help has no such effect (16). Social activities, group activities, fitness exercise, and intellectual participation can all reduce the risk of depression in older persons, but helping and dedication activities have no significant effect (17). Voluntary participation has positive effects but obligatory participation has harmful influences (18). In addition, some studies suggested that SP could relieve depression among certain older adults (19–21). However, most studies were conducted using ordinary least squares (OLS) regression, and a few of them use propensity score matching, which could reduce bias and improve internal validity of the statistical analysis. In addition, few studies have explored the correlation between depression and SP in China while considering frequency, type, and quantity of SP. This study made the following assumptions: first, SP was associated with a lower depression score. The higher the frequency of SP, the larger the quantity of SP, and the more diverse the types of SP, the lower the depression score.

Our study explored the impact of SP on depression among middle-aged and older Chinese persons using a PSM method and explored the relationships with respect to the frequency, type, and quantity of SP. Effects were further compared among different age groups, genders, and places of residence. The findings could provide evidence for the targeted social participation measures for specific groups of middle-aged and older people.

## Methods

### Sample and Data Collection

The datasets were obtained from 2018 wave of the China Health and Retirement Longitudinal Study, a national representative survey adopting a four-stage, stratified, cluster sampling method to collect Chinese residents aged 45 years and above, covering 450 villages and 150 counties in 28 provinces in China, and the detailed sampling methods can be retrieved from the study of Zhao et al. (22). The baseline survey started in 2011 (first wave) and conducted follow-up surveys every 2–3 years, and the 2018 wave is the latest wave data. In the 2018 CHARLS database, 19,816 observations were admitted from the 2018 CHARLS database. After screening and excluding invalid samples, a total of 9,312 respondents were included in the study, Supplementary Figure S1 shows the process of sample screening.

### Variables

#### Outcome Variable

Depression is the outcome variable in this study, which is estimated by the CES-D10 scale. The respondents were asked the frequency of their feeling or behavior during the last week, and each answer of positively oriented questions was coded from 0 to 3 according to the frequent value; the answers of the negatively oriented questions were reverse-coded. The total score of the scale ranges from 0 to 30, with higher scores indicating more serious depression. Our study used the score of 12 as the cutoff point to describe the prevalence of depression (23). In the previous related studies, CES-D 10 showed a good internal reliability (24).

#### Explanatory Variable

The explanatory variable in this study is SP, which was estimated by their participation in 11 social activities in the past month. If respondents participated in any of the social activities, they were considered to have SP, otherwise, they were considered not to have SP.

#### Confounding Variables

The following variables were selected as possible confounding variables according to previous studies (21, 25–27). Confounding variables include three aspects: demographic characteristics, including gender, age, marital status, education, retirement, residency, individual income, medical insurance, number of family member, and living near child/children; lifestyle characteristics, including smoking, physical activity, and alcohol consumption; and health status, including types of NCDs, self-rated health changes. Detail codes, and definitions are shown in Table 1.

**Table 1:** Definition/codes of the variables.

**Variable**	**Code/definition**
Gender	0 = man; 1 = woman
Age	Continuous variable
Marital status	0 = single; 1 = partnered
Education	1 = Illiterate; 2 = primary school; 3 = middle school; 4 = high school
Residency	0 = rural; 1 = urban
Retirement	0 = no; 1 = yes
Individual financial situation	Individual income annually, continuous variable
Physical activity	Took physical activity at least 10 min every week. 0 = no; 1 = yes
Alcohol consumption	Ever consumed any alcohol last year. 0 = no; 1 = yes
Smoke	Ever chewed tobacco, smoked a pipe, or smoked cigarette last year. 0 = no; 1 = yes
Medical insurance	Type(s) of participating health insurance 1 = Urban employee medical insurance 2 = Urban and rural resident medical insurance (integrated urban resident medical insurance and new rural cooperative medical insurance) 3 = Urban resident medical insurance 4 = New rural cooperative medical insurance 5 = participating in any kind of social insurance and private insurance at the same time 6 = No insurance or other types of insurance
Living near child(ren)	0 = Living in other places or living abroad 1 = Living together or living in the same/neighboring courtyard
Number of family members	The number of family members living together and sharing family income and expenses with respondents, continuous variable
Numbers of NCDs	14-item summaries of any physical noncommunicable diseases, including hypertension, dyslipidemia, diabetes, cancer, chronic lung diseases, liver disease, heart attack, stroke, kidney diseases, stomach or other digestive diseases, memory-related disease, emotional, nervous, or psychiatric problems, arthritis or rheumatism, and asthma 1 = none; 2 = 1 type; 3 = 2 types; 4 = ≥3 types
Self-rated health changes	Health status compared with last wave in 2015. 1 = better; 2 = about the same; 3 = worse
Depression	Applying CES-10 scale to estimate depression, continuous variable
SP	Participating in any activities in 13 items (interacting with friends/mah-jong or cards/sports or social clubs/community-related organizations/voluntary or charity work/using Internet/providing help/cared for a sick or disabled adult/attended an educational or training course/tocks investment/other) 0 = no; 1 = yes
SP frequent(s)	The maximum frequency of SP 1 = none; 2 = not regularly; 3 = ≥1/week
SP variety	Cumulative number of participants in 13 activities 1 = none; 2 = 1 type; 3 ≥2 types

### Statistical Analysis

A chi-square test was used on categorical variables to test the differences in social participation and its diversity and frequency. In addition, an independent sample *t*-test was used for continuous variables to assess social participation differences, an analysis of variance (ANOVA) was performed to evaluate the differences in social participation's diversity and frequency.

PSM was used to estimate the effect of SP on depression. As the data we used were observational, there existed the risk of bias caused by study design, unbalanced distribution or grouping, nonrandom sampling, subjective tendency of the measurer, and so on, as well as confounding variables, all of which can lead to biased estimation (28). PSM can address covariate imbalances between treatment and control groups in observational studies by matching respondents with similar characteristics in the two groups. This can reduce the influence of data bias and confounding variables and facilitate a more reasonable comparison between the exposure and control groups. Observational data are thereby rendered more akin to random trial data, and robust estimation results may be obtained (29–31). Thus, this study used the PSM method to estimate the average treatment effect of SP on depression of the middle-aged and older people.

The PSM method was first proposed by Rosenbaum and Rubin (32), and it has become one of the important empirical methods to deal with nonrandom data. The process of the PSM method is as follows: first, calculating propensity scores by logit regression model to predict the probability of SP, which was matched by confounding variables. We then calculated the average treatment effect for the treated (ATT), and the formula is as follows:


$$\begin{array}{l} ATT=E\left[\left(h_{i1}|SP_{i}=1\right)\right]-E\left[\left(h_{i0}|SP_{i}=1\right)\right] \end{array}$$

where *E*[(*h*_*i*1_|*SP*_*i*_ = 1)] represents the depression scores of respondents participating in social activities, and *E*[(*h*_*i*0_|*SP*_*i*_ = 1)] is the reference outcome.

Next, the matching effect was assessed by testing the absolute standard bias (ASB) between the treatment and control groups after matching. Previous studies showed that the smaller the ASB of confounding variables after matching, the better is the matching effect (32). According to Rosenbaum and Rubin's definition, if ASB is <20, the matching effect can be considered reliable (32). We used three matching methods including K-nearest neighbor matching (1:1), radius matching, and kernel matching to ensured the robustness of our results. In the radius matching, a 0.2 SD caliper was employed, and in the kernel matching, a bandwidth of 0.06 was set with an epan kernel.

In addition, we also applied ordinary least squares (OLS) regression models to estimate the impact of SP on depression, and we used robust standard errors to alleviate the potential effect of heteroscedasticity. All statistical analyses were conducted by STATA version MP17.0 software. A *p*-value < 0.05 was considered as statistically significant.

## Results

### Characteristics of Respondents

The characteristics of respondents are shown in Table 2. These include the characteristics of social participation and its diversity and frequency among the respondents. Most respondents were women (51.91%), partnered (80.00%), rural residents (71.09%), and with a median age of 64 years old. Of the respondents, the education level was illiterate, primary school, middle school, and ≥high school, which accounted for 17.72, 44.50, 23.38, and 14.40%, respectively. The median of the depression score was 8, the value for the SP and no SP groups were 7 and 9, respectively, and the prevalence of depression symptoms among middle-aged and older Chinese people was 42.92%. Whether middle-aged and older people engaged in social participation differed with respect to most characteristics: social participation was associated with younger respondents, those who were not of single marital status, with better economic status, living in an urban area, having at least a high school education, and living far from child/children. The frequency and variety of social participation differed with respect to most of the characteristics.

**Table 2:** Basic information of respondents.

	**Total** **(*N* = 9,312)**	**SP**		***p***		**Variety**			***P***		**Frequency**			***P***
		**No**	**Yes**			**None** **(*N* = 4,052)**	**1 type** **(*N* = 2,820)**	**≥2 types** **(*N* = 2,440)**			**None** **(*N* = 4,052)**	**Not regularly** **(*N* = 1,345)**	**≥1/week** **(*N* = 3,915)**	
	***n* (%)**	***n* (%)**	***n* (%)**			***n* (%)**	***n* (%)**	***n* (%)**			***n* (%)**	***n* (%)**	***n* (%)**	
**Gender**				0.069					<0.001					0.043
Man	4,478 (48.09)	1,992 (44.48)	2,486 (55.52)			1,992 (44.48)	1,253 (27.98)	1,233 (27.53)			1,992 (44.48)	663 (14.81)	1,823 (40.71)	
Woman	4,834 (51.91)	2,060 (42.61)	2,774 (57.39)			2,060 (42.61)	1,567 (32.42)	1,207 (24.97)			2,060 (42.61)	682 (14.11)	2,092 (43.28)	
**Age**				<0.001					<0.001					<0.001
<60	3,459 (37.15)	1,236 (35.73)	2,223 (64.27)			1,236 (35.73)	1,051 (30.38)	1,172 (33.88)			1,236 (35.73)	552 (15.96)	1,671 (48.31)	
60–74	4,255 (45.69)	1,996 (46.91)	2,259 (53.09)			1,996 (46.91)	1,258 (29.57)	1,001 (23.53)			1,996 (46.91)	614 (14.43)	1,645 (38.66)	
≥75	1,598 (17.16)	820 (51.31)	778 (48.69)			820 (51.31)	511 (31.98)	267 (16.71)			820 (51.31)	179 (11.20)	599 (37.48)	
Median (min, max)	64 (45, 111)	66 (45, 90)	63 (45, 111)	<0.001		66 (45, 98)	64 (45, 98)	60 (45, 111)	<0.001		66 (45, 98)	63 (46, 92)	63 (45, 111)	<0.001
**Marital status**				0.001					<0.001					0.001
Single	1,862 (20.00)	874 (46.94)	988 (53.06)			874 (46.94)	599 (32.17)	389 (20.89)			874 (46.94)	231 (12.41)	757 (40.66)	
Partnered	7,450 (80.00)	3,178 (42.66)	4,272 (57.34)			3,178 (42.66)	2,221 (29.81)	2,051 (27.53)			3,178 (42.66)	1,114 (14.95)	3,158 (42.39)	
**Education**				<0.001					<0.001					<0.001
Illiterate	1,650 (17.72)	933 (56.55)	717 (43.45)			933 (56.55)	516 (31.27)	201 (12.18)			933 (56.55)	194 (11.76)	523 (31.70)	
≤Primary school	4,144 (44.50)	1,968 (47.49)	2,176 (52.51)			1,968 (47.49)	1,299 (31.35)	877 (21.16)			1,968 (47.49)	643 (15.52)	1,533 (36.99)	
Middle school	2,177 (23.38)	814 (37.39)	1,363 (62.61)			814 (37.39)	660 (30.32)	703 (32.29)			814 (37.39)	337 (15.48)	1,026 (47.13)	
≥high school	1,341 (14.40)	337 (25.13)	1,004 (74.87)			337 (25.13)	345 (25.73)	659 (49.14)			337 (25.13)	171 (12.75)	833 (62.12)	
**Residency**				<0.001					<0.001					<0.001
Rural	6,620 (71.09)	3,178 (48.01)	3,442 (51.99)			3,178 (48.01)	2,026 (30.60)	1,416 (21.39)			3,178 (48.01)	988 (14.92)	2,454 (37.07)	
Urban	2,692 (28.91)	874 (32.47)	1,818 (67.53)			874 (32.47)	794 (29.49)	1,024 (38.04)			874 (32.47)	357 (13.26)	1,461 (54.27)	
**Retirement**				0.120					0.009					<0.001
No	5,976 (64.18)	2,636 (44.11)	3,340 (55.89)			2,636 (44.11)	1,745 (29.20)	1,595 (26.69)			2,636 (44.11)	922 (15.43)	2,418 (40.46)	
Yes	3,336 (35.82)	1,416 (42.45)	1,920 (57.55)			1,416 (42.45)	1,075 (32.22)	845 (25.33)			1,416 (42.45)	423 (12.68)	1,497 (44.87)	
**Individual income annually**				<0.001					<0.001					<0.001
Median (min, max)	0 (0, 1,280,000)	0 (0, 530,000)	0 (0, 1,280,000)			0 (0, 530,000)	0 (0, 438,000)	0 (0, 530,000)			0 (0, 530,000)	0 (0, 400,000)	0 (0, 530,000)	
**Physical activity**				<0.001					0.407					<0.001
No	6,373 (68.44)	2,765 (43.39)	3,608 (56.61)			2,765 (43.39)	1,956 (30.69)	1,652 (25.92)			2,765 (43.39)	831 (13.04)	2,777 (43.57)	
Yes	2,939 (31.56)	1,287 (43.79)	1,652 (56.21)			1,287 (43.79)	864 (29.40)	788 (26.81)			1,287 (43.79)	514 (17.49)	1,138 (38.72)	
**Alcohol consumption**				<0.001					<0.001					<0.001
No	6,129 (65.82)	2,895 (47.23)	3,234 (52.77)			2,895 (47.23)	1,877 (30.62)	1,357 (22.14)			2,895 (47.23)	821 (13.40)	2,413 (39.37)	
Yes	3,183 (34.18)	1,157 (36.35)	2,026 (63.65)			1,157 (36.35)	943 (29.63)	1,083 (34.02)			1,157 (36.35)	524 (16.46)	1,502 (47.19)	
**Smoke**				<0.001					0.063					0.015
No	6,938 (74.51)	3,029 (43.66)	3,909 (56.34)			3,029 (43.66)	2,132 (30.73)	1,777 (25.61)			3,029 (43.66)	960 (13.84)	2,949 (42.51)	
Yes	2,374 (25.49)	1,023 (43.09)	1,351 (56.91)			1,023 (43.09)	688 (28.98)	663 (27.93)			1,023 (43.09)	385 (16.22)	966 (40.69)	
**Medical insurance**				<0.001					<0.001					<0.001
UEBMI	1,308 (14.05)	363 (27.75)	945 (72.25)			363 (27.75)	386 (29.51)	559 (42.74)			363 (27.75)	177 (13.53)	768 (58.72)	
URBMI	1,120 (12.03)	504 (45.00)	616 (55.00)			504 (45.00)	366 (32.68)	250 (22.32)			504 (45.00)	159 (14.20)	457 (40.80)	
NCMS	386 (4.15)	152 (39.38)	234 (60.62)			152 (39.38)	129 (33.42)	105 (27.20)			152 (39.38)	45 (11.66)	189 (48.96)	
Integrated insurance	5,710 (61.32)	2,768 (48.48)	2,942 (51.52)			2,768 (48.48)	1,729 (30.28)	1,213 (21.24)			2,768 (48.48)	864 (15.13)	2,078 (36.39)	
Mixture	338 (3.63)	64 (18.93)	274 (81.07)			64 (18.93)	83 (24.56)	191 (56.51)			64 (18.93)	45 (13.31)	229 (67.75)	
Other types and none	450 (4.83)	201 (44.67)	249 (55.33)			201 (44.67)	127 (28.22)	122 (27.11)			201 (44.67)	55 (12.22)	194 (43.11)	
**Living near child (ren)**				<0.001					<0.001					0.001
No	3,951 (42.43)	1,634 (41.36)	2,317 (58.64)			1,634 (41.36)	1,152 (29.16)	1,165 (29.49)			1,634 (41.36)	589 (14.91)	1,728 (43.74)	
Yes	5,361 (57.57)	2,418 (45.10)	2,943 (54.90)			2,418 (45.10)	1,668 (31.11)	1,275 (23.78)			2,418 (45.10)	756 (14.10)	2,187 (40.79)	
**Number of family members**				0.313					0.533					0.587
Median (min, max)	0 (0, 9)	0 (0, 8)	0 (0, 9)			0 (0, 8)	0 (0, 9)	0 (0, 7)			0 (0, 8)	0 (0, 6)	0 (0, 9)	
**Numbers of NCDs**				0.364					0.543					0.634
None	5,117 (54.95)	2,269 (44.34)	2,848 (55.66)			2,269 (44.34)	1,525 (29.80)	1,323 (25.85)			2,269 (44.34)	717 (14.01)	2,131 (41.65)	
1 type	2,612 (28.05)	1,110 (42.50)	1,502 (57.50)			1,110 (42.50)	821 (31.43)	681 (26.07)			1,110 (42.50)	383 (14.66)	1,119 (42.84)	
2 types	974 (10.46)	415 (42.61)	559 (57.39)			415 (42.61)	295 (30.29)	264 (27.10)			415 (42.61)	150 (15.40)	409 (41.99)	
≥3 types	609 (6.54)	258 (42.36)	351 (57.64)			258 (42.36)	179 (29.39)	172 (28.24)			258 (42.36)	95 (15.60)	256 (42.04)	
**Self-rated health changes**				<0.001					<0.001					<0.001
Better	756 (8.12)	296 (39.15)	460 (60.85)			296 (39.15)	246 (32.54)	214 (28.31)			296 (39.15)	114 (15.08)	346 (45.77)	
About the same	3,890 (41.77)	1,568 (40.31)	2,322 (59.69)			1,568 (40.31)	1,174 (30.18)	1,148 (29.51)			1,568 (40.31)	577 (14.83)	1,745 (44.86)	
Worse	4,666 (50.11)	2,188 (46.89)	2,478 (53.11)			2,188 (46.89)	1,400 (30.00)	1,078 (23.10)			2,188 (46.89)	654 (14.02)	1,824 (39.09)	
**Depression**				<0.001					<0.001					<0.001
No	5,315 (57.08)	2,149 (40.43)	3,166 (59.57)			2,149 (40.43)	1,604 (30.14)	1,564 (29.43)			2,149 (40.43)	764 (14.37)	2,402 (45.19)	
Yes	3,997 (42.92)	1,903 (47.61)	2,094 (52.39)			1,903 (47.61)	1,218 (30.47)	876 (21.92)			1,903 (47.61)	581 (14.54)	1,513 (37.85)	
Median (min, max)	8 (0, 30)	9 (0, 30)	7 (0, 30)	<0.001		9 (0, 30)	8 (0, 30)	7 (0, 30)	<0.001		9 (0, 30)	8 (0, 30)	7 (0, 30)	<0.001

### Social Participation Rate of Respondents With Different Characteristics

**Figure 1:**
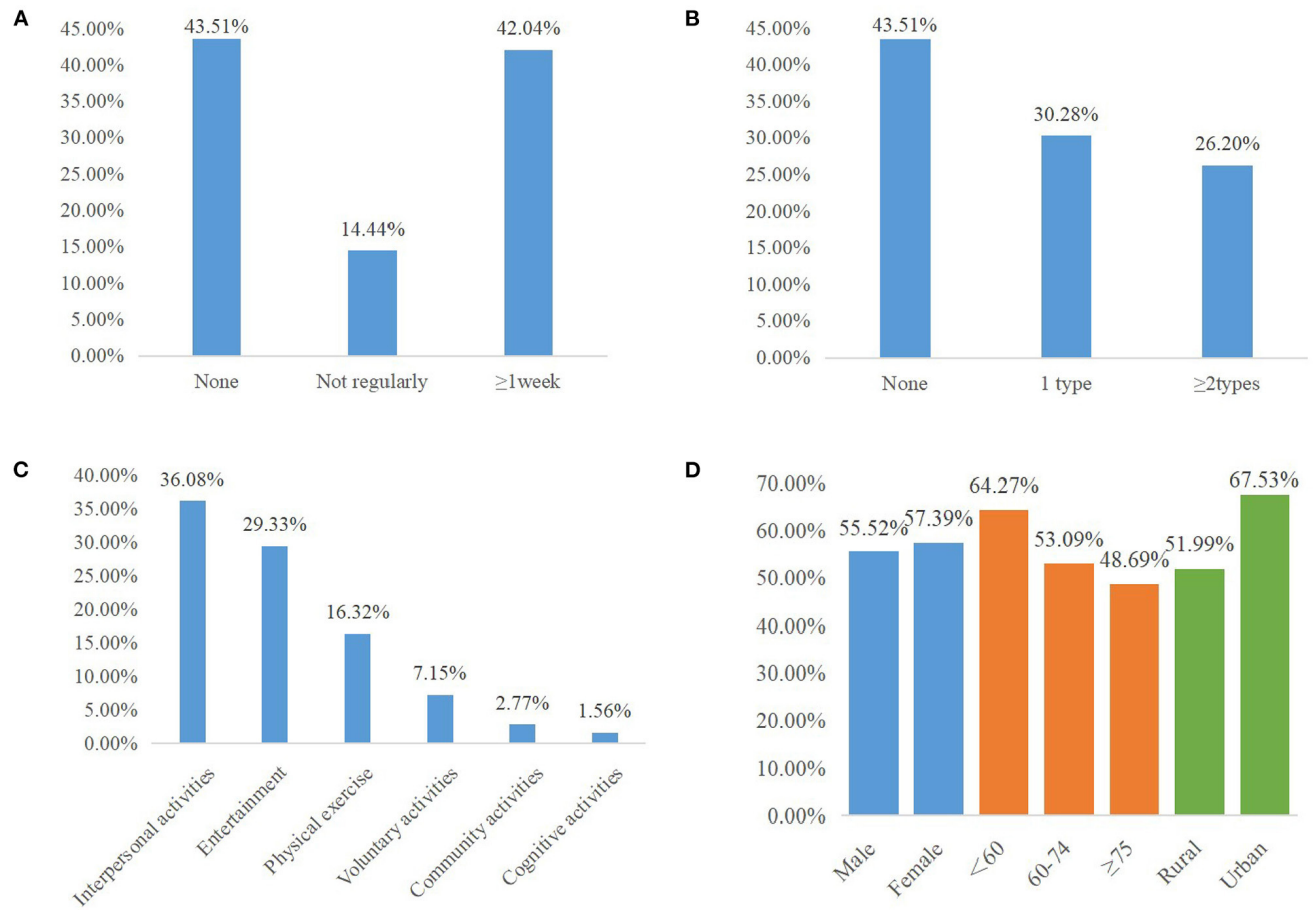
Social participation rate of respondents with different frequencies **(A)**, numbers **(B)**, types **(C)**, and social participation rate by different ages, genders, and residencies **(D)**.

Figure 1 shows the social participation rate of respondents with different types (Figure 1C), numbers (Figure 1B), and frequencies (Figure 1A) and social participation rate by different ages, genders, and residencies (Figure 1D). The proportion of social participation among middle-aged and older people account for 56.49%, 30.28% of the respondents participated in one activity, and 26.20% participated in at least two activities, 42.04% of the respondents participated in activities more than one time a week. We also obtained that among all types of social participation, the top two types with the highest participation rate are interpersonal activities with 36.08% and entertainment with 29.33%; significant differences were shown among different types, numbers, and frequencies of social participation. The social participants' rate had no significant difference between men and women, while respondents aged <60 years, or those in urban regions, had a higher participation rate than the reference group.

### The Probability of Social Participation Among Middle-Aged and Older People

The estimation results of the probability having SP among middle-aged and older people by logit model are depicted in Supplementary Table S1 (Supplementary File 1). Through the logit model, the PSM score was estimated, and the matching of treatment group and control group was completed. The finding showed that pseudo-R2 is 0.0540, indicating that the model fits well, and the association between possible confounding variables and social participation was revealed, for example, those younger (β = −0.021, *p* < 0.001) and those with better self-rated health changes (β = −0.179, *p* < 0.001) were more likely to have social participation.

### Effect Test on Depression Using Unmatched and Matched Data

**Figure 2:**
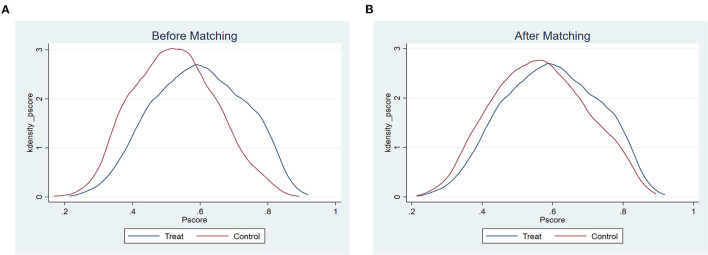
Density function of propensity scores before **(A)** and after **(B)** PSM.

**Figure 3:**
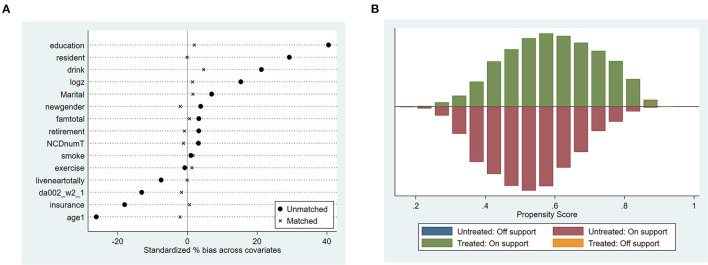
Covariates' balancing test (standard bias of the covariates **(A)** and common support hypothesis histogram **(B)**).

After data matching, we conducted a series of tests to check the matching effect. First, we compared the density function of propensity scores of the treated group and control group. Figure 2 shows that the distribution of the two groups is closer than before matching. Next, the covariates' balancing test was conducted. The results are depicted in Figure 3. All the standard bias of the covariates are reduced substantially after matching (Figure 3A). The covariates showed no significant difference among the two groups in Supplementary Tables S2–S4 (Supplementary File 1), and all the ASB are <20%, which indicates that the matching has a good quality. We constructed a histogram of the propensity score in the treated and control group (Figure 3B), as Figure 3B shows no essential difference in the distribution of propensity score of the treatment group and the control group after matching. It was clear that no matter which matching method we used, the propensity score distribution became symmetrical. Most of the observations are in the expected range, thus, satisfying the common support hypothesis.

### Propensity Score Matching Estimation of the Effect of Social Participation on Depression Scores

Table 3 reports the estimation results before and after PSM. Overall, the significance and magnitude of the effect of SP on depression scores all decreased. After PSM, the results suggested that SP had a positive effect on decreasing depression scores (*p* < 0.001) by 0.875–0.898 points, namely, SP was associated with lower depression scores. Taking the result of the radius matching method as an example, the depression scores of the respondents with SP were 0.881 points (reduction rate was 9.20%) lower, on average, than those of persons without SP. All types of SP had a significantly positive effect on decreasing depression scores (*p* < 0.001); of the different types of SP, participating in community activities had the largest effect (−1.549 to −1.788). PSM estimation also showed that compared with no SP, taking part in one or two types of social activity had significant negative effects on depression scores, while the more activities participants engaged in, the lower their depression scores. PSM estimation suggested that a higher frequency of participation promoted lower depression scores.

**Table 3:** Propensity score matching (PSM) estimation results for the effect of social participation (SP) on depression scores.

**Variable**	**Method**	**Treated**	**Controls**	**ATT**	**Standard error**	***t***
**SP**						
	Unmatched	8.690	10.095	−1.404^**^	0.141	−9.950
	*K*-nearest neighbor matching (1:1)	8.696	9.571	−0.875^**^	0.209	−4.190
	Radius matching	8.696	9.577	−0.881^**^	0.162	−5.430
	Kernel matching	8.696	9.594	−0.898^**^	0.160	−5.610
**Types of SP**						
**Interpersonal activities** ^**a**^						
	Unmatched	8.753	10.095	−1.342^**^	0.160	−8.390
	*K*-nearest neighbor matching (1:1)	8.753	9.675	−0.922^**^	0.236	−3.900
	Radius matching	8.753	9.730	−0.977^**^	0.178	−5.490
	Kernel matching	8.753	9.739	−0.986^**^	0.176	−5.620
**Physical exercise** ^**b**^						
	Unmatched	7.799	10.095	−2.296^**^	0.289	−7.940
	*K*-nearest neighbor matching (1:1)	7.872	9.608	−1.737^**^	0.427	−4.070
	Radius matching	7.872	9.275	−1.403^**^	0.312	−4.500
	Kernel matching	7.872	9.307	−1.435^**^	0.308	−4.650
**Voluntary activities** ^**c**^						
	Unmatched	8.652	10.094	−1.443^**^	0.208	−6.940
	*K*-nearest neighbor matching (1:1)	8.662	9.264	−0.602^*^	0.305	−1.970
	Radius matching	8.662	9.408	−0.746^**^	0.233	−3.20
	Kernel matching	8.662	9.415	−0.752^*^	0.229	−3.280
**Community activities** ^**d**^						
	Unmatched	6.888	10.095	−3.207^**^	0.448	−7.160
	*K*-nearest neighbor matching (1:1)	6.984	8.533	−1.549^*^	0.604	−2.570
	Radius matching	6.984	8.706	−1.723^**^	0.429	−4.010
	Kernel matching	6.984	8.772	−1.788^**^	0.425	−4.200
**Entertainment** ^**e**^						
	Unmatched	7.873	10.095	−2.222^**^	0.165	−13.430
	*K*-nearest neighbor matching (1:1)	7.881	9.096	−1.215^**^	0.267	−4.550
	Radius matching	7.881	9.008	−1.127^**^	0.208	−5.420
	Kernel matching	7.881	9.001	−1.120^**^	0.205	−5.460
**Cognitive activities** ^**f**^						
	Unmatched	6.145	10.095	−3.950^**^	0.591	−6.680
	*K*-nearest neighbor matching (1:1)	6.183	7.345	−1.162	0.876	−1.330
	Radius matching	6.113	7.635	−1.522^*^	0.727	−2.090
	Kernel matching	6.183	7.760	−1.577^*^	0.697	−2.260
**Numbers of different types of SP**						
1 type	Unmatched	9.254	10.095	−0.840^**^	0.169	−4.960
	*K*-nearest neighbor matching (1:1)	9.262	9.854	−0.592^*^	0.232	−2.550
	Radius matching	9.262	9.922	−0.660^**^	0.172	−3.830
	Kernel matching	9.262	9.946	−0.684^**^	0.172	−3.980
≥2 types	Unmatched	8.039	10.095	−2.056^**^	0.173	−11.880
	*K*-nearest neighbor matching (1:1)	8.038	9.213	−1.175^**^	0.286	−4.110
	Radius matching	8.038	9.182	−1.143^**^	0.218	−5.240
	Kernel matching	8.038	9.194	−1.156^**^	0.213	−5.440
**Frequency of SP**						
Not regularly	Unmatched	9.140	10.095	−0.955^**^	0.220	−4.350
	*K*-nearest neighbor matching (1:1)	9.145	9.891	−0.746^*^	0.305	−2.450
	Radius matching	9.145	9.781	−0.636^**^	0.222	−2.860
	Kernel matching	9.145	9.796	−0.651^**^	0.221	−2.950
≥1/week	Unmatched	8.536	10.095	−1.559^**^	0.151	−10.310
	*K*-nearest neighbor matching (1:1)	8.543	9.378	−0.836^**^	0.233	−3.580
	Radius matching	8.543	9.513	−0.971^**^	0.175	−5.540
	Kernel matching	8.543	9.529	−0.986^**^	0.173	−5.700

a *Interpersonal activities, including interacting with friends*.

b *Physical exercise, including sport or social clubs*.

c *Voluntary activity, including voluntary or charity work, providing help or caring for a sick or disabled adult*.

d *Community activities, including community-related organizations*.

e *Entertainment, including mah-jong or cards, using the Internet*.

f *Cognitive activities, including stock investment or attending an educational or training course*.

* *p < 0.05*.

** *p < 0.01*.

*ATT, average treatment effect for the treated*.

### Estimation Results for the Effect of Social Participation on Depression Scores Using Ordinary Least Squares

The results estimated using OLS are shown in Supplementary Table S5 (Supplementary File 1). In model 1, SP had a significant negative effect on depression scores (coefficient = −1.404, *p* < 0.001). Control variables related to demographic characteristic variables were added in Model 2, further lifestyle characteristics were added in Model 3, and health status characteristics were added in Model 4; the coefficient of SP increased to −0.892, −0.868, and −0.774, respectively, while the regression significance level remained at the 1% level. The regression results imply that compared with middle-aged and older adults who did not engage in SP, those who participated in social activities exhibited a depression score that was reduced by 0.63, on average, after adjusting for the control variables.

### Heterogeneity Analysis of the Effect of Social Participation on Depression

We further conducted a heterogeneity analysis by age, gender, and residency (Supplementary Tables S6–S8 in Supplementary File 1). The results showed that SP had a significant negative effect on depression scores among different ages, genders, and residencies: being female woman, aged ≥75, and living in an urban area were all associated with more negative effects on depression scores.

## Discussion

This study applied a PSM method to explore the effect of SP on depression scores, including examining the effect with respect to the type, number, and frequency of SP, and further conducted a heterogeneity analysis. In addition, we also applied OLS regression models to estimate the impact of SP on depression. Our main findings indicated that SP was associated with significantly reduced depression scores: participating in community activities, higher frequency of SP, and engaging in more types of SP promoted a lower depression score. In the subgroup analysis, SP had a greater effect among older persons, women, and urban residents. The results using OLS also revealed a negative impact of SP on depression scores.

### Prevalence of Depression Symptoms in Middle-Aged and Older Chinese People

Through this large-scale investigation, we found that the prevalence of depression among middle-aged and older Chinese people was 42.92%. Although the CESD-10 scale was widely used in depression investigation, it was just the estimation of depression symptom, not obsoletely equal to the real diagnosis and, thus, highly far from the monitoring data. In domestic studies using the CESD-10 scale to measure depression, a study showed that the prevalence of depression of the middle-aged and older in 2015 was 32.62% (33). Zhou found that depression prevalence was 35.19% in 2018 (34), which can be seen that the trend of prevalence is rising and the recent finding was close to us. An explanation was that physical pain caused by chronic diseases often leads to mental disorder, which may lead to depressive symptoms. Domestic studies showed that middle-aged and older people are not only the main patients with chronic diseases but also the high-risk groups of depression (35).

Nevertheless, the result in our research was also higher than other previous studies that used an estimated scale; the incidence of depression is not only higher than in earlier reports (25, 36, 37), but also exceeds that of other developed countries (19, 38, 39), and even some developing countries (40–42). The high prevalence is a reminder that depression in Chinese middle-aged and older persons is an urgent concern. China will face greater challenges in terms of the increasing burden of depression; accordingly, attention must be paid to this issue, and feasible preventive measures are urgently needed.

### Social Participation Could Benefit Older Middle-Aged and Older Adults' Mental Health

Our study verified that SP promotes mental health. We found that respondents who engaged in SP had lower depression scores, consistent with previous studies (18, 43–45). This could be explained by social participation incentivizing mutual support, providing a sense of belonging, and largely reducing social isolation (46, 47), which, therefore, result in better mental health (48). Due to aging, retirement, and declining health quality, among other factors, middle-aged and older people may experience a considerable psychological burden, social isolation, changed social roles, and the need to adapt to aging; these factors may promote depression. Against a background of population aging trends worldwide, mental health has attracted substantial research attention. The WHO noted out that SP is a key factor in healthy aging (49). Therefore, effective measures should be developed to promote SP. Policy makers should provide support for middle-aged and older people, first, by constructing service facilities suitable for this population, and second, forming related organizations with a community or village committee as a unit and encouraging middle-aged and older people to actively participate in social activities.

### Participating in Community Activities, Higher Frequency of Social Participation, and More Types of Social Participation Promoted Lower Depression Scores

We proceed to explore the associations between depression and different types of social engagement. We observed that taking part in community activities was the best way to promote mental health among all types of activities considered. Community activities are carried out by a mutually beneficial organization formed by people with certain common characteristics; we speculate that such organizations are associated with promoting a sense of belonging. However, the participation rate of community activities was only 2.77%. In China, development of leagues for older persons is incomplete and informal; most leagues are self-organized and lack legal status. Consequently, there is no legal basis for the construction and operation of associations for older persons and, hence, a lack of guarantee in terms of policies, organizations, and funds. This results in many contradictions, difficulties, and problems in the construction of associations for older persons (50). In addition, we observed that engaging in more diverse social activities and engaging in SP more than once a week were relatively conducive to mental health, consistent with prior studies (14, 25, 51). Therefore, governments should make effective use of the community as a platform to promote the development of diverse activities, such as by providing financial support. Furthermore, incentive mechanisms could be used to promote the participation of middle-aged and older people in various activities.

### Social Participation Had a Greater Effect on Older Persons, Women, and Urban Residents

Through further comparing the effect of subgroups of age, gender, and residency, we determined that SP had a greater effect on women, and urban residents. We also found that the older the age, the greater the impact. People over 75 years of age had the largest effect among the age groups. The percentage of social participants had no significant difference between men and women, while respondents aged <75 years, or those in urban regions, had a higher value than the reference group. The above results emphasize that SP appears to be a more effective measure to prevent depression for women, older persons, and urban residents.

Older women are more likely to lack social interaction than older men; furthermore, influenced by the traditional Chinese concept of “men dominate the outside, women dominate the interior” (52), older men often enter the labor market to earn income and have frequent contact with the outside world, while older women generally engage in housework or take care of children or older relatives at home, compared with men. This part of communication with the outside world is insufficient for women. Activities in which women participated were the primary source of relief for their depressive symptoms and, thus, had a more considerable effect on them.

As age grows, the mental health status and social adaptability of the elderly generally decreased, and the possibility of social isolation become higher (53). Our study also showed that as age increased, social participation decreased; thus, social participation plays a greater role among the venerable age group. Residents of urban regions received more benefit than did rural residents, as the former had a more convenient environment for participation, due to greater availability of diverse social activities in a city. Targeted measures should consider women, elderly, and rural residents, such as middle-aged and older women, and elderly persons with limited mobility. For example, accessibility of participation should be improved by installing elevators in buildings or introducing social workers to conduct activities in the homes of elderly people with disabilities. For rural elderly persons, the social activities currently available have limited effects on improving health. Activities, such as “guangchangwu” (after-dinner community square dancing) and other community activities that are widespread in cities have beneficial effects on health, are rarely available in rural areas (54). Therefore, it is necessary to improve the provision of various organized activities for elderly persons in rural areas and to guide such persons to participate in these activities on a consistent basis.

### Comparison of Effects Between Propensity Score Matching and Ordinary Least Squares Analysis Strategies

A significant negative association between SP and depression scores was found in both PSM and OLS analysis, although the estimated effect was larger in the OLS method than the PSM method, that is, not balancing covariates led to overestimation of the treatment effect.

The primary strength of our study is that the PSM method enabled a reasonable comparison to be made between groups with and without SP, reducing estimation bias. There are also some limitations to our study. First, as a cross-sectional study, causal relationships between SP and depression could not be determined; future studies should use longitudinal designs to address this limitation. Second, PSM cannot solve endogenous problems, such as self-selection and omission of variables, which may affect the accuracy of the results. Third, we obtained the result from scale responses, thus, it reflects a degree of mental status, namely, depressive symptoms. However, it does not equate to depression clinically diagnosed by a physician. Fourth, the sample size of persons who took part in several types of SP, such as voluntary activities, community organizations, or using the Internet, was small; using a larger sample of such persons would provide more reliable results, future studies are needed for such further exploration.

## Conclusion

This study highlighted the crucial role of SP in preventing depression and promoting the mental health of middle-aged and older Chinese people. Community activities were the most effective of all activities. Participation in more diverse activities and participation with the frequencies of once a week or more is beneficial to mental health. SP had a greater effect on older persons, women, and urban residents. The results suggest that as the population of China ages, the government should provide support for middle-aged and older person's social participation, such as by providing venues, funds, and channels for social participation. Organized and diverse activities should be provided and measures should be taken to encourage middle aged and older persons participated in such social engagement. In particular, attention should be paid to women, rural residents, and older persons, who would benefit from targeted measures provided according to their needs.

## Data Availability Statement

The dataset are available from the CHARLS repository, http://charls.pku.edu.cn.

## Ethics Statement

This study was approved by the Ethical Committees of Peking University. During the investigation, all participants provided written informed consent. CHARLS is publicly available and de-identified. The patients/participants provided their written informed consent to participate in this study.

## Author Contributions

JW and QW designed the study. JW contributed to the data processing and drafted the manuscript. JW, JX, YN, and PP contributed to the result analysis. XZ and SS contributed to the collection of literature and review of the manuscripts. YL, HL, LL, and LG assisted by providing suggestions for this manuscript. QW and YH revised the paper. All authors approved the current version of this manuscript for publication. All authors contributed to the article and approved the submitted version.

## Funding

This study was funded by the National Social Science Fund of China (Grant No. 19AZD013) and the National Natural Science Foundation of China (Grant No. 71804036).

## Conflict of Interest

The authors declare that the research was conducted in the absence of any commercial or financial relationships that could be construed as a potential conflict of interest.

## Publisher's Note

All claims expressed in this article are solely those of the authors and do not necessarily represent those of their affiliated organizations, or those of the publisher, the editors and the reviewers. Any product that may be evaluated in this article, or claim that may be made by its manufacturer, is not guaranteed or endorsed by the publisher.
