# Supplementary material for: Effects of Social Participation and Its Diversity, Frequency, and Type on Depression in Middle-Aged and Older Persons: Evidence From China
Source: Front Psychiatry. 2022 Apr 25;13:825460. doi: 10.3389/fpsyt.2022.825460 (PMC9085245; doi:10.3389/fpsyt.2022.825460)
Supplement: Supplementary file 1 [file Data_Sheet_1.doc]

Table S1 The probability of social participation of middle-aged and old people by logit model.

|  | β | 95%CI | |
| --- | --- | --- | --- |
|  |  | Upper | Lower |
| Gender | 0.445**^**^** | 0.332 | 0.557 |
| Age | -0.021**^**^** | -0.026 | -0.015 |
| Marital status | -0.077 | -0.193 | 0.039 |
| Education | 0.333**^**^** | 0.279 | 0.387 |
| Retirement | 0.185**^**^** | 0.072 | 0.297 |
| Residency | 0.315**^**^** | 0.202 | 0.427 |
| Log (individual income annually) | 0.008 | -0.003 | 0.018 |
| Smoke | 0.135**^*^** | 0.021 | 0.249 |
| Physical activity | 0.090 | -0.010 | 0.190 |
| Alcohol consumption | 0.485**^**^** | 0.383 | 0.587 |
| Medical Insurance | -0.055**^*^** | -0.091 | -0.019 |
| Number of family members | 0.000 | -0.063 | 0.064 |
| Types of NCDs | 0.084**^**^** | 0.035 | 0.132 |
| Living near child(ren) | -0.100**^*^** | -0.194 | -0.005 |
| Self-rated health changes | -0.179**^**^** | -0.247 | -0.110 |
| Constant | 0.800**^*^** | 0.264 | 1.335 |
| Pseudo-R^2^ | 0.0540 | | |
| Number of observations | 9312 | | |

***p<0.05; **p<0.01**

Table S2 Covariate balancing using the nearest-neighbor matching method

|  | Sample | Mean | | Bias (%) | Bias reduction (%) | t | P-value |
| --- | --- | --- | --- | --- | --- | --- | --- |
|  |  | Treated | Control |  |  |  |  |
| Gender | Unmatched | 0.527 | 0.508 | 3.8 |  | 1.82 | 0.069 |
|  | Matched | 0.527 | 0.535 | -1.6 | 57.9 | -0.82 | 0.412 |
| Age | Unmatched | 63.551 | 66.087 | -26.1 |  | -12.52 | <0.001 |
|  | Matched | 63.570 | 63.420 | 1.5 | 94.1 | 0.81 | 0.420 |
| Marital | Unmatched | 0.812 | 0.784 | 6.9 |  | 3.33 | 0.001 |
|  | Matched | 0.812 | 0.802 | 2.6 | 63.1 | 1.34 | 0.182 |
| Education | Unmatched | 2.505 | 2.137 | 40.5 |  | 19.24 | <0.001 |
|  | Matched | 2.502 | 2.496 | 0.6 | 98.4 | 0.32 | 0.750 |
| Retirement | Unmatched | 0.365 | 0.349 | 3.2 |  | 1.55 | 0.121 |
|  | Matched | 0.365 | 0.369 | -0.8 | 74.3 | -0.43 | 0.671 |
| Residency | Unmatched | 0.346 | 0.216 | 29.2 |  | 13.85 | <0.001 |
|  | Matched | 0.345 | 0.368 | -5.4 | 81.7 | -2.55 | 0.011 |
| Log (individual income annually) | Unmatched | 3.108 | 2.403 | 15.3 |  | 7.28 | <0.001 |
|  | Matched | 3.104 | 3.002 | 15.3 | 85.5 | 1.09 | 0.274 |
| Smoke | Unmatched | 0.257 | 0.252 | 1.0 |  | 0.48 | 0.631 |
|  | Matched | 0.257 | 0.242 | 3.4 | -239.4 | 1.76 | 0.079 |
| Physical activity | Unmatched | 0.314 | 0.318 | -0.8 |  | -0.37 | 0.715 |
|  | Matched | 0.314 | 0.304 | 2.2 | -189.4 | 1.14 | 0.254 |
| Alcohol consumption | Unmatched | 0.385 | 0.286 | 21.2 |  | 10.10 | <0.001 |
|  | Matched | 0.384 | 0.371 | 2.9 | 86.2 | 1.45 | 0.147 |
| Medical Insurance | Unmatched | 3.329 | 3.560 | -18.0 |  | -8.53 | <0.001 |
|  | Matched | 3.332 | 3.360 | -2.2 | 88.0 | -1.04 | 0.296 |
| Number of family members | Unmatched | 0.531 | 0.506 | 3.3 |  | 1.56 | 0.228 |
|  | Matched | 0.530 | 0.518 | 1.5 | 52.7 | 0.78 | 0.438 |
| Types of NCDs | Unmatched | 1.698 | 1.670 | 3.2 |  | 1.51 | 0.132 |
|  | Matched | 1.698 | 1.685 | 1.5 | 53.9 | 0.74 | 0.461 |
| Living near child(ren) | Unmatched | 0.560 | 0.597 | -7.5 |  | -3.61 | <0.001 |
|  | Matched | 0.559 | 0.548 | 2.3 | 69.8 | 1.16 | 0.247 |
| Self-rated health changes | Unmatched | 2.384 | 2.467 | -13.1 |  | -6.27 | <0.001 |
|  | Matched | 2.385 | 2.396 | -1.6 | 87.4 | -0.84 | 0.402 |
| **Sample** | Pseudo R^2^ | LR chi^2^ | p > chi^2^ | Mean Bias | R | Var (%) |  |
| Unmatched | 0.054 | 691.79 | <0.001 | 12.9 | 1.32 | 43 |  |
| Matched | 0.002 | 26.38 | 0.034 | 2.2 | 0.99 | 29 |  |

***p<0.05; **p<0.01**

Table S3 Covariate balancing using the radius matching method

| Variable | Sample | Mean | | Bias (%) | Bias reduction (%) | t | P-value |
| --- | --- | --- | --- | --- | --- | --- | --- |
|  |  | Treated | Control |  |  |  |  |
| Gender | Unmatched | 0.527 | 0.508 | 3.8 |  | 1.82 | 0.069 |
|  | Matched | 0.527 | 0.539 | -2.5 | 34.9 | -1.27 | 0.204 |
| Age | Unmatched | 63.551 | 66.087 | -26.1 |  | -12.52 | ＜0.001 |
|  | Matched | 63.570 | 63.747 | -1.8 | 93.0 | -0.96 | 0.339 |
| Marital | Unmatched | 0.812 | 0.784 | 6.9 |  | 3.33 | 0.001 |
|  | Matched | 0.812 | 0.806 | 1.7 | 75.9 | 0.87 | 0.382 |
| Education | Unmatched | 2.505 | 2.137 | 40.5 |  | 19.24 | ＜0.001 |
|  | Matched | 2.502 | 2.496 | 0.7 | 98.2 | 0.36 | 0.718 |
| Retirement | Unmatched | 0.365 | 0.349 | 3.2 |  | 1.55 | 0.121 |
|  | Matched | 0.365 | 0.372 | -1.4 | 56.4 | -0.72 | 0.471 |
| Residency | Unmatched | 0.346 | 0.216 | 29.2 |  | 13.85 | ＜0.001 |
|  | Matched | 0.345 | 0.351 | -1.4 | 95.1 | -0.69 | 0.492 |
| Log (individual income annually) | Unmatched | 3.108 | 2.403 | 15.3 |  | 7.28 | ＜0.001 |
|  | Matched | 3.104 | 3.041 | 1.4 | 91.0 | 0.68 | 0.499 |
| Smoke | Unmatched | 0.257 | 0.252 | 1.0 |  | 0.48 | 0.631 |
|  | Matched | 0.257 | 0.249 | 1.9 | -90.6 | 0.98 | 0.326 |
| Physical activity | Unmatched | 0.314 | 0.318 | -0.8 |  | -0.37 | 0.715 |
|  | Matched | 0.314 | 0.306 | 1.8 | -129.9 | 0.90 | 0.366 |
| Alcohol consumption | Unmatched | 0.385 | 0.286 | 21.2 |  | 10.10 | ＜0.001 |
|  | Matched | 0.384 | 0.364 | 4.2 | 80.1 | 2.10 | 0.036 |
| Medical Insurance | Unmatched | 3.329 | 3.560 | -18.0 |  | -8.53 | ＜0.001 |
|  | Matched | 3.332 | 3.312 | 1.6 | 91.2 | 0.77 | 0.443 |
| Number of family members | Unmatched | 0.531 | 0.506 | 3.3 |  | 1.56 | 0.118 |
|  | Matched | 0.530 | 0.525 | 0.7 | 77.1 | 0.38 | 0.704 |
| Types of NCDs | Unmatched | 1.698 | 1.670 | 3.2 |  | 1.51 | 0.132 |
|  | Matched | 1.698 | 1.708 | -1.2 | 62.6 | -0.60 | 0.552 |
| Living near child(ren) | Unmatched | 0.560 | 0.597 | -7.5 |  | -3.61 | ＜0.001 |
|  | Matched | 0.559 | 0.558 | 0.4 | 95.3 | 0.18 | 0.857 |
| Self-rated health changes | Unmatched | 2.384 | 2.467 | -13.1 |  | -6.27 | ＜0.001 |
|  | Matched | 2.385 | 2.393 | -1.3 | 90.1 | -0.66 | 0.510 |
| **Sample** | Pseudo R^2^ | LR chi^2^ | p > chi^2^ | Mean Bias | R | Var (%) |  |
| Unmatched | 0.054 | 691.79 | 0.000 | 12.9 | 1.32 | 43 |  |
| Matched | 0.000 | 7.11 | 0.955 | 1.6 | 1.00 | 14 |  |

***p<0.05; **p<0.01**

Table S4 Covariate balancing using the kernel matching method

| Variable | Sample | Mean | | Bias (%) | Bias reduction (%) | t | P-value |
| --- | --- | --- | --- | --- | --- | --- | --- |
|  |  | Treated | Control |  |  |  |  |
| Gender | Unmatched | 0.527 | 0.508 | 3.8 |  | 1.82 | 0.069 |
|  | Matched | 0.527 | 0.537 | -2.1 | 44.7 | -1.08 | 0.281 |
| Age | Unmatched | 63.551 | 66.087 | -26.1 |  | -12.52 | ＜0.001 |
|  | Matched | 63.570 | 63.776 | -2.1 | 91.9 | -1.11 | 0.265 |
| Marital | Unmatched | 0.812 | 0.784 | 6.9 |  | 3.33 | 0.001 |
|  | Matched | 0.812 | 0.806 | 1.5 | 77.8 | 0.81 | 0.420 |
| Education | Unmatched | 2.505 | 2.137 | 40.5 |  | 19.24 | ＜0.001 |
|  | Matched | 2.502 | 2.485 | 2.0 | 95.2 | 0.96 | 0.335 |
| Retirement | Unmatched | 0.365 | 0.349 | 3.2 |  | 1.55 | 0.121 |
|  | Matched | 0.365 | 0.369 | -0.9 | 73.4 | -0.44 | 0.660 |
| Residency | Unmatched | 0.346 | 0.216 | 29.2 |  | 13.85 | ＜0.001 |
|  | Matched | 0.345 | 0.345 | -0.1 | 99.6 | -0.05 | 0.960 |
| Log (individual income annually) | Unmatched | 3.108 | 2.403 | 15.3 |  | 7.28 | ＜0.001 |
|  | Matched | 3.104 | 3.039 | 1.4 | 90.8 | 0.70 | 0.486 |
| Smoke | Unmatched | 0.257 | 0.252 | 1.0 |  | 0.48 | 0.631 |
|  | Matched | 0.257 | 0.250 | 1.6 | -58.4 | 0.82 | 0.414 |
| Physical activity | Unmatched | 0.314 | 0.318 | -0.8 |  | -0.37 | 0.715 |
|  | Matched | 0.314 | 0.308 | 1.3 | -69.3 | 0.67 | 0.505 |
| Alcohol consumption | Unmatched | 0.385 | 0.286 | 21.2 |  | 10.10 | ＜0.001 |
|  | Matched | 0.384 | 0.362 | 4.7 | 78.1 | 2.32 | 0.021 |
| Medical Insurance | Unmatched | 3.329 | 3.560 | -18.0 |  | -8.53 | ＜0.001 |
|  | Matched | 3.332 | 3.325 | 0.6 | 96.8 | 0.28 | 0.779 |
| Number of family members | Unmatched | 0.531 | 0.506 | 3.3 |  | 1.56 | 0.118 |
|  | Matched | 0.530 | 0.526 | 0.6 | 82.8 | 0.29 | 0.774 |
| Types of NCDs | Unmatched | 1.698 | 1.670 | 3.2 |  | 1.51 | 0.132 |
|  | Matched | 1.698 | 1.708 | -1.1 | 64.2 | -0.57 | 0.569 |
| Living near child(ren) | Unmatched | 0.560 | 0.597 | -7.5 |  | -3.61 | ＜0.001 |
|  | Matched | 0.559 | 0.560 | -0.1 | 98.9 | -0.04 | 0.966 |
| Self-rated health changes | Unmatched | 2.384 | 2.467 | -13.1 |  | -6.27 | ＜0.001 |
|  | Matched | 2.385 | 2.396 | -1.7 | 86.8 | -0.88 | 0.379 |
| **Sample** | Pseudo R^2^ | LR chi^2^ | p > chi^2^ | Mean Bias | R | Var (%) |  |
| Unmatched | 0.054 | 691.79 | 0.000 | 12.9 | 1.32 | 43 |  |
| Matched | 0.001 | 7.85 | 0.930 | 1.4 | 1.06 | 14 |  |

***p<0.05; **p<0.01**

Table S5 The effect of social participation on CES-D 10 score

|  | Model1 | | | Model2 | | | Model3 | | | Model4 | | |
| --- | --- | --- | --- | --- | --- | --- | --- | --- | --- | --- | --- | --- |
|  | β | 95%CI | | β | 95%CI | | β | 95%CI | | β | 95%CI | |
|  |  | lower | upper |  | lower | upper |  | lower | upper |  | lower | upper |
| Social participant | -1.404** | -1.684 | -1.125 | -0.892** | -1.171 | -.612 | -0.868** | -1.148 | -0.588 | -0.774** | -1.044 | -0.505 |
| **Demographic characteristics** |  |  |  |  |  |  |  |  |  |  |  |  |
| Gender |  |  |  | 1.756** | 1.468 | 2.044 | 1.862** | 1.519 | 2.204 | 1.928** | 1.600 | 2.256 |
| Age |  |  |  | -0.039** | -0.057 | -0.022 | -0.036** | -0.054 | -0.018 | -0.037** | -0.054 | -0.020 |
| Marital status |  |  |  | -0.863** | -1.238 | -0.488 | -0.854** | -1.228 | -0.479 | -0.870** | -1.228 | -0.513 |
| Education |  |  |  | -0.946** | -1.106 | -0.785 | -0.906** | -1.067 | -0.745 | -0.829** | -0.983 | -0.675 |
| Residency |  |  |  | -1.267** | -1.607 | -0.927 | -1.133** | -1.476 | -0.790 | -1.045** | -1.372 | -0.717 |
| Retirement |  |  |  | 0.690** | 0.337 | 1.044 | 0.854** | 0.492 | 1.217 | 0.592** | 0.246 | 0.937 |
| Log (individual income annually) |  |  |  | -0.093** | -0.124 | -0.061 | -0.087** | -0.119 | -0.056 | -0.071** | -0.101 | -0.040 |
| Medical Insurance |  |  |  | 0.225** | 0.121 | 0.330 | 0.215** | 0.110 | 0.319 | 0.230** | 0.130 | 0.330 |
| Living near child(ren) |  |  |  | -0.107 | -0.401 | 0.187 | -0.093 | -0.386 | 0.201 | -0.051 | -0.333 | 0.231 |
| Number of family members |  |  |  | -0.262* | -0.458 | -0.066 | -0.276* | -0.471 | -0.081 | -0.242* | -0.433 | -0.051 |
| **Lifestyle characteristics** |  |  |  |  |  |  |  |  |  |  |  |  |
| Physical activity |  |  |  |  |  |  | 0.669** | 0.362 | 0.976 | 0.564** | 0.268 | 0.860 |
| Alcohol consumption |  |  |  |  |  |  | -0.581** | -0.881 | -0.281 | -0.416* | -0.704 | -0.128 |
| Smoke |  |  |  |  |  |  | 0.623** | 0.289 | 0.957 | 0.664** | 0.342 | 0.985 |
| **Physical health** |  |  |  |  |  |  |  |  |  |  |  |  |
| Types of NCDs |  |  |  |  |  |  |  |  |  | 1.025** | 0.874 | 1.175 |
| Self-rated health changes |  |  |  |  |  |  |  |  |  | 2.244** | 2.041 | 2.447 |
| N | 9312 |  |  | 9312 |  |  | 9312 |  |  | 9312 |  |  |
| Pseudo R^2^ | 0.0105 |  |  | 0.0912 |  |  | 0.0953 |  |  | 0.1674 |  |  |

**Adjustments: Model 1: none; Model 2: adjust for gender, age, marital status, education, residency, retirement, individual income annually, medical Insurance, living near child(ren), number of family members; Model 3: further adjust for physical activity, alcohol consumption, smoke; Model 4：further adjust for types of NCDs, Self-rated health changes**

***p<0.05; **p<0.01**

Table S6 PSM estimation results for the effect of social participation on depression score by age

| Variable | Method | ATT | Standard error | t |
| --- | --- | --- | --- | --- |
| Age<60 |  |  |  |  |
| Depression | Unmatched | -1.206** | 0.233 | -5.180 |
|  | K-nearest neighbor matching | -0.647 | 0.359 | -1.800 |
|  | Radius matching | -0.637* | 0.273 | -2.330 |
|  | Kernel matching | -0.642* | 0.268 | -2.400 |
| Age between 60-74 |  |  |  |  |
| Depression | Unmatched | -1.419** | 0.212 | -6.710 |
|  | K-nearest neighbor matching | -1.094** | 0.314 | -3.480 |
|  | Radius matching | -0.914** | 0.239 | -3.830 |
|  | Kernel matching | -0. 934** | 0.237 | -3.950 |
| Age ≥75 |  |  |  |  |
| Depression | Unmatched | -1.620** | 0.341 | -4.75 |
|  | K-nearest neighbor matching | -1.072* | 0.482 | -2.23 |
|  | Radius matching | -1.207** | 0.370 | -3.26 |
|  | Kernel matching | -1.271** | 0.365 | -3.48 |

***p<0.05; **p<0.01**

Table S7 PSM estimation results for the effect of social participation on depression score by gender

| Variable | Method | Male | | |  | Female | | |
| --- | --- | --- | --- | --- | --- | --- | --- | --- |
|  |  | ATT | Standard error | t |  | ATT | Standard error | t |
| Depression | Unmatched | -1.328** | 0.185 | -7.180 |  | -1.570** | 0.204 | -7.700 |
|  | K-nearest neighbor matching | -0.924** | -0.288 | -3.21 |  | -1.220** | 0.313 | -3.900 |
|  | Radius matching | -0.840** | 0.218 | -3.850 |  | -0.876** | 0.236 | -3.720 |
|  | Kernel matching | -0.833** | 0.215 | -3.870 |  | -0.895** | 0.231 | -3.880 |

***p<0.05; **p<0.01**

Table S8 PSM estimation results for the effect of social participation on depression score by residency

| Variable | Method | Rural | | |  | Urban | | |
| --- | --- | --- | --- | --- | --- | --- | --- | --- |
|  |  | ATT | Standard error | t |  | ATT | Standard error | t |
| Depression | Unmatched | -0.906** | 0.169 | -5.380 |  | -1.848** | 0.259 | -7.130 |
|  | K-nearest neighbor matching | -0.644** | 0.239 | -2.700 |  | -1.059* | 0.429 | -2.470 |
|  | Radius matching | -0.695** | 0.178 | -3.890 |  | -1.213** | 0.325 | -3.730 |
|  | Kernel matching | -0.710** | 0.177 | -4.010 |  | -1.199** | 0.320 | -3.750 |

***p<0.05; **p<0.01**


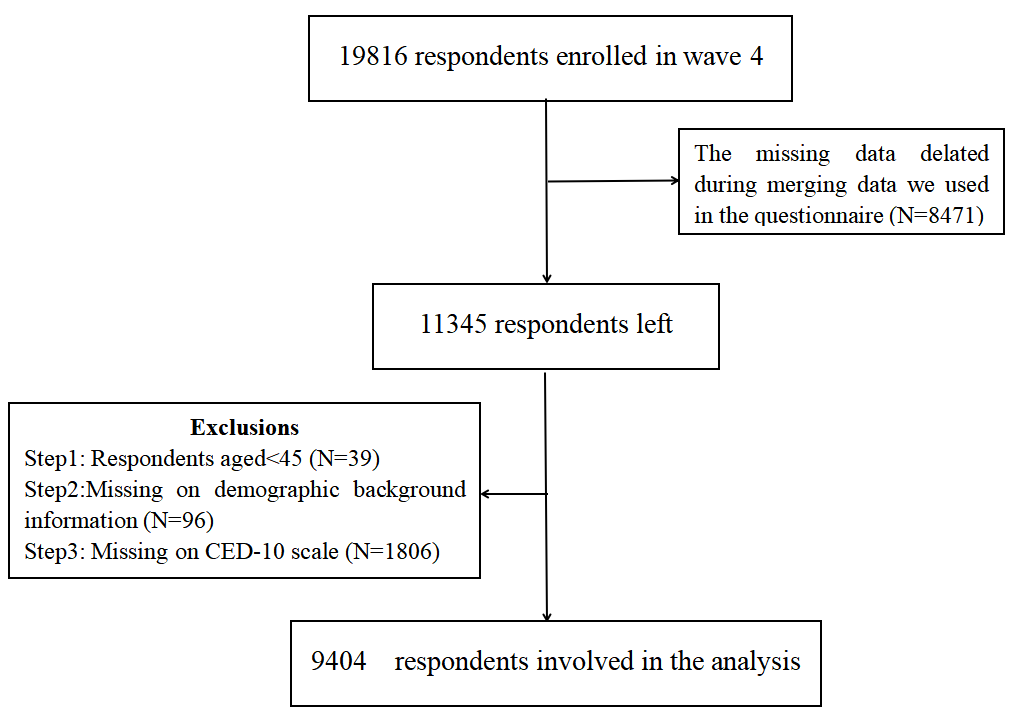


**Figure S1 The flowchart of participant selection.**
